# On Some Novel Modes of Relief in Certain Visual Defects

**Published:** 1878-10

**Authors:** H. Gradle


### ON SOME NOVEL MODES OF RELIEF IN CERTAIN VISUAL DEFECTS.

By H. Gradle.

*(Paper read before the Chicago Medical Society, August 5, 1878.)*

Gentlemen: My remarks of this evening pertain to a case of conical cornea under my care some months ago. The patient, a lawyer, some 42 years of age, gave the usual history of this complaint, viz: gradually increasing indistinctness of sight since puberty. Both eyes appeared as if a drop of dew rested upon the corneæ. Their conical shape was very apparent, especially in profile view, while the irregularity of curvature of the surface showed itself in the distortion of the image of the window reflected from the cornea. The apex of each cornea was slightly clouded; beyond this, however, the optic media were perfectly transparent. The ophthalmoscopic examination in the direct image was impossible, on account of the irregular refraction. The inverted image, although not sharp, indicated no disease of the interior of the eye, which was, besides, excluded by a perfect field of vision and a perfect recognition of colors.

On account of the normal peripheral vision and the gradual accustomation to his present state, the patient could walk safely even on crowded streets. He was, however, unable to recognize human features at any distance. Although the increased curvature of the cornea would naturally put the eye into a myopic condition, the irregularity in refraction was so considerable that sight was not better proportionately for objects close by, than for the distance. He could count fingers with his left eye at five feet distance, with his right eye at three feet. The cause of the visual indistinctness is readily understood. Since the exact focussing of rays of light entering the pupil depends in the first place on a proper curvature of the cornea, the irregularity of its surface in the present instance prevented, of course, any reunion of the rays into a single focus. When the rays of light are brought to a focus in front of or behind the retina, as in the ordinary anomalies of refraction, we can bring the focus to its proper place upon the retina by the use of correcting glasses. But in the present instance the rays were not at all united into a focal point, but scattered irregularly, and hence no spherical or cylindrical glass could be of much avail. This unpleasant but logical deduction had in fact been confirmed by previous patient, but unsuccessful attempts by other oculists. However, by shutting off the larger number of rays by the use of a narrow diaphragm, the retinal images gained in distinctness, but at the expense of the illumination. Through such a narrow opening he could count fingers at 15, and with the other eye at 10 feet distance. In fact the improvement obtained was sufficient to warrant use of a pair of opaque spectacles, with central perforation for a closer examination of distant objects, although their use on the streets would have been dangerous, on account of the narrowing of the field of vision. Close by, however, he saw no better with them, since the increased distinctness was too highly paid for by the impaired illumination. With the naked eye he could recognize large letters at the distance of one or two inches, but this was even more difficult when using a diaphragm. Neither the dilatation of the pupil by atropine, nor its contraction by eserine changed the visual acuity to any extent.

I proposed to the patient an operation, either the ablation of the conical apex after v. Graefe's original method, or the trephining of the cornea as practiced by Bowman and Wecker. But although a good result, i. e., a return of the cornea to its normal curvature, is often obtained, a failure of the operation is by no means infrequent. The patient hence declined any surgical procedure for the present, but willingly undertook with me some optical experiments.

The refraction, which light undergoes when passing through the cornea may be expressed as the product of two factors, on the one hand the curvature of the surface, on the other hand the difference between the refractive power of the corneal tissue and of the surrounding medium, in the present case the air. Since the index of refraction of the cornea differs very little from that of water, the optical function of the cornea ceases when the eye is submerged under water. Hence we find the cornea of fishes flat, since any curvature whatever would be useless.

Physiologists (especially Czermak) have sometimes used an instrument called the orthoscope, for some optical researches. This consists of a frame, pressed watertight against the rim of the orbit with a front wall of glass, while the interspace between the cornea and the glass is filled with water. I had previously made some experiments with this apparatus at the clinic of Dr. Meyer, in Paris, but did not finish them on account of my departure. Instead of water, I made use of a fluid resembling the composition of the tears, viz : a $\frac{1}{2}$ per cent solution of sodium chloride with a slight addition of gum acacia, but after all found that tepid water was equally well borne. In order to render the whole water tight, I lined the frame with a rubber tube, to serve as an air cushion. On thus submerging the cornea under water, the light passing through the water is not deviated from its course when it enters the cornea whatever be its curvature, and hence the focussing of the optic system depends on the curvature of the glass wall of the apparatus. I made use of a plane glass, but mounted in front of it the proper lens, in the present case a convex glass of 20 dioptrics (about 2 inch focus).

With these means the patient enjoyed, for the first time since his youth, a sight almost normal. At twenty feet distance he read with ease type No. 40 of Snellen's tables, while with an additional glass, convex 6, Jaeger's brilliant type (No. 1.) was read fluently (the eye being atropinized). The apparatus caused neither pain nor inconvenience to the eye, at least when in place for half an hour. But its permanent use was forbidden by a different obstacle. The pressure necessary to prevent any leaking between the frame and the skin could not be endured for any length of time without injuring the skin, and during the few days the patient remained in town I could neither obtain nor invent any practicable contrivance.

Finding this device impracticable, I began investigating how the usefulness of a narrow diaphragm could be increased. Theory and experiment showed that the object must be: (1.) strongly illuminated (to counteract the darkening produced by the diaphragm), and (2.) brought close to the eye (because on approaching the object, its retinal image increases in size in a greater proportion than the circles of dispersion.) Both indications could be fulfilled by examining, not the object itself, but its inverted image in an astronomical telescope. By employing an objective lens of large aperture, the illumination of the image could be increased to almost any extent, its distance from the eye being of course dependent on the strength of the objective, while the proper ocular, with a narrow diaphragm, permitted an accurate perception of the image formed close to the eye.

On combining various lenses at my disposal on this principle, he saw plainly in the distance (V. $\frac{20}{30}$), and with the proper focussing read newspaper type (of course when inverted.) Reading, however, was not perfectly easy, on account of the difficulty of following the lines, and the magnifying of every tremor of the hand, still he read nearly as fluently as a normal observer. The patient, unduly particular as to appearances, hesitated however to order an instrument, none being found ready in the market, since he disliked the unavoidable inverting of the objects.

The principles, however, above mentioned, could be also utilized in the case of an ordinary terrestrial telescope, though to a less extent, but with the advantage of seeing objects erect. A small field-glass with narrow diaphragm over the ocular improved his distant vision materially, while the addition of a convex six in front of the objective, permitted at least the reading of papers.

Similar experiments can undoubtedly improve sight materially, in the more numerous cases of irregular astigmatism, in fact in any visual defect, dependant on irregular curvature of the corneal surface.

